# Proteomics based markers of clinical pain severity in juvenile idiopathic arthritis

**DOI:** 10.1186/s12969-022-00662-1

**Published:** 2022-01-15

**Authors:** Hanne Van Der Heijden, Benoit Fatou, Diana Sibai, Kacie Hoyt, Maria Taylor, Kin Cheung, Jordan Lemme, Mariesa Cay, Benjamin Goodlett, Jeffery Lo, Melissa M. Hazen, Olha Halyabar, Esra Meidan, Rudy Schreiber, Camilo Jaimes, Kirsten Ecklund, Lauren A. Henderson, Margaret H. Chang, Peter A. Nigrovic, Robert P. Sundel, Hanno Steen, Jaymin Upadhyay

**Affiliations:** 1 Department of Anesthesiology, Critical Care and Pain Medicine, Boston Children’s Hospital, Harvard Medical School, Boston, MA USA; 2 Faculty of Psychology and Neuroscience, Section Neuropsychology & Psychopharmacology Maastricht University, Maastricht, The Netherlands; 3 Faculty of Science, Biomedical Sciences Neurobiology, University of Amsterdam, Amsterdam, The Netherlands; 4 Department of Pathology, Boston Children’s Hospital, Harvard Medical School, Boston, MA USA; 5 Division of Immunology, Boston Children’s Hospital, Harvard Medical School, Boston, MA USA; 6 BioSAS Consulting, Inc, Wellesley, MA USA; 7 Division of Genetics and Genomics, Boston Children’s Hospital, Harvard Medical School, Boston, MA USA; 8 Department of Radiology, Boston Children’s Hospital, Harvard Medical School, Boston, MA USA; 9 Neurobiology Program, Boston Children’s Hospital, Boston, MA USA; 10 Precision Vaccines Program, Boston Children’s Hospital, Boston, MA USA; 11 Department of Psychiatry, McLean Hospital, Harvard Medical School, MA Belmont, USA

**Keywords:** Proteomics, Mass spectrometry, Juvenile idiopathic arthritis, Pain, Inflammation

## Abstract

**Introduction:**

Juvenile idiopathic arthritis (JIA) is a cluster of autoimmune rheumatic diseases occurring in children 16 years of age or less. While it is well-known that pain may be experienced during inflammatory and non-inflammatory states, much remains ambiguous regarding the molecular mechanisms that may drive JIA pain. Thus, in this pilot study, we explored the variability of the serum proteomes in relation to pain severity in a cohort of JIA patients.

**Methods:**

Serum samples from 15 JIA patients (male and female, 12.7 ± 2.8 years of age) were assessed using liquid chromatography/mass spectrometry (LC/MS). Correlation analyses were performed to determine the relationships among protein levels and self-reported clinical pain severity. Additionally, how the expression of pain-associated proteins related to markers of inflammation (Erythrocyte Sedimentation Rate (ESR)) or morphological properties of the central nervous system (subcortical volume and cortical thickness) implicated in JIA were also evaluated.

**Results:**

306 proteins were identified in the JIA cohort of which 14 were significantly (*p* < 0.05) associated with clinical pain severity. Functional properties of the identified pain-associated proteins included but were not limited to humoral immunity (IGLV3.9), inflammatory response (PRG4) and angiogenesis (ANG). Associations among pain-associated proteins and ESR (IGHV3.9, PRG4, CST3, VWF, ALB), as well as caudate nucleus volume (BTD, AGT, IGHV3.74) and insular cortex thickness (BTD, LGALS3BP) were also observed.

**Conclusions:**

The current proteomic findings suggest both inflammatory- and non-inflammatory mediated mechanisms as potential factors associated with JIA pain. Validation of these preliminary observations using larger patient cohorts and a longitudinal study design may further point to novel serologic markers of pain in JIA.

**Supplementary Information:**

The online version contains supplementary material available at 10.1186/s12969-022-00662-1.

## Abstract (article highlights)

In the current preliminary study, serum proteins associated with pain severity were identified in JIA patients. Pain-associated proteins had functional roles predominantly in immune or inflammatory processes.

Correlations of pain-associated proteins with ESR and altered CNS morphology were also observed with ESR and altered CNS morphology were also observed.

Larger-scale as well as longitudinal studies are needed to determine if evaluation of the proteome may provide a platform for identifying novel analgesic targets in JIA.

**Supplementary Information:**

The online version contains supplementary material available at 10.1186/s12969-022-00662-1.

## Introduction

Juvenile idiopathic arthritis (JIA) is a common childhood rheumatic illness categorized into seven subtypes and characterized by musculoskeletal joint pathology present for greater than 6 weeks [1, 2]. One of the most frequently occurring and debilitating symptoms of JIA is pain [3, 4], significantly impacting quality of life [5]. Pain in JIA appears to have multifactorial causes and can be driven by varying mechanisms [6]. Pain, particularly when induced by inflammation, may be ameliorated by pharmacological approaches such as disease modifying anti-rheumatic drugs (DMARDs) and biologics (e.g., tumor necrosis factor (TNF)-α inhibitors). Non-pharmacological modalities, including exercise or cognitive behavioral therapy, also may be prescribed in parallel [4, 7–13]. Notwithstanding the availability of these therapeutic options, mitigating pain in JIA remains challenging, which may in part stem from unknown, non-inflammatory pain processes that are active in some JIA patients.Cytokine products secreted by macrophages and T cells due to an activated immune system, are considered to mediate a chronic inflammatory status and joint pathology in JIA [14, 15]. For example, levels of interleukin (IL) 18 in serum and synovial fluid of JIA patients have been identified as marker of disease severity, and TNF-α, macrophage inhibitory factor (MIF), IL-1, IL-6 and members of the CC chemokine family have been reported to contribute to inflammatory responses in JIA [15–17]. Moreover, several autoantibodies are currently used to distinguish JIA subtypes. Yet, no biomarkers have been validated for the purposes of guiding pain treatment in JIA populations [18]. To this end, we have embarked on a liquid-chromatography/mass spectrometry (LC/MS)-based serum proteomic approach, where protein expression in complex fluid samples is identified and accurately quantified [19–21]. A proteomic approach previously led to identification of proteins associated with ion channels, receptors and signaling pathways implicated with acute and chronic pain states [22]. Furthermore, MS has previously been utilized to decipher protein composition in cerebrospinal fluid in patients with fibromyalgia and rheumatoid arthritis (RA) [23]. Although the synovial proteome in JIA patients has been investigated in prior work [24, 25], to our knowledge, a serum proteomics approach in JIA in the context of pain has not been carried out as of now.

The aim of this preliminary study was to identify pain-associated proteins in JIA to gain further insight into the biological underpinnings of pain in this rheumatic condition, which in turn may set the foundation for identifying novel therapeutic targets for pain treatment. In the current report, an LC/MS-based proteomics analysis of blood serum samples was performed to identify proteins that associate with clinical pain severity in a cohort of JIA patients. Subsequently, the association was examined between pain-associated proteins and other aspects of JIA, such as erythrocyte sedimentation rate (ESR), clinical juvenile arthritis disease activity score (cJADAS) [26], and CNS morphological properties previously implicated in JIA [27].

## Methods

### Study participants

Male and female JIA patients (*N* = 15, 12.5 ± 2.8 years) were evaluated in this study (see also **Supplemental Fig.** **1**). This cohort was described in our prior report [27]. Patients were recruited from the Rheumatology Program at Boston Children’s Hospital (BCH), following approval of the BCH Institutional Review Board. Written consent and assent were provided by the patients’ parent or guardian and patient, respectively before participation in this investigation. Prior to blood sample collection (2 mL), patients completed the PROMIS® Numeric Rating Scale (0–10 scale), which is a self-assessment of clinical pain over the last 7 days (PROMIS; http://www.healthmeasures.net). In this scale, 0 corresponds to no pain and 10 is the worst pain imaginable. The cJADAS, specifically the cJADAS-10, a composite disease activity score for JIA, which does not include an acute phase reactant, was determined for each patient at the time of enrollment [26]. Blood sample collection and completion of the clinical pain rating questionnaire were accomplished during the same study visit. One patient did not provide a blood sample. All enrolled patients were on active treatment and underwent clinical examination to determine the presence of pain and other elements of arthritis, including joint inflammation, redness, stiffness, or tenderness. Furthermore, JIA patients were also evaluated with non-contrast MRI to detect possible bone erosion, cartilage degradation, joint space narrowing, bone marrow edema, soft-tissue edema, joint fluid, synovitis, and tenosynovitis. Each musculoskeletal MRI dataset was assessed by a board-certified pediatric radiologist (Dr. Ecklund).’ ESR values were available for 13 out of the 16 patients, while combined neuroimaging and musculoskeletal MRI analyses were performed as previously described in nine out of the 16 patients [27].

### LC/MS analysis

A volume of 1ul serum was first diluted in 60 μl of urea buffer (8 M in 50 mM ammonium bicarbonate buffer) and then 15 μl of dithiothreitol (0.05 M final concentration) was added and the samples were incubated 30 min at 800 rpm in room temperature. A volume of 10 μl of iodoacetamide was added and an incubation was performed for 30 min at 800 rpm in room temperature in dark. A volume of 10ul of 0.05 M DTT was added to quench the alkylation and the sample was incubated 15 min at room temperature. The samples were then transferred to SP3 beads mixture (1:10 protein to beads) [28], previously washed twice with HPLC water. A volume of 150ul of absolute ethanol was added to the sample/bead mixture before incubation at 1000 rpm at room temperature for 10 min. The mixture was then transferred to an automatic liquid handling robot instrument (Opentrons, NY) to wash the beads and remove the supernatant before adding 1 μg of trypsin and incubating for 2 h at 37 °C at 1000 rpm on thermomixer. After trypsin digestion, the samples were centrifuged 10 min at 3220×g before acidification using 2% v/v formic acid.

A discovery-based proteomic workflow was performed with no depletion nor fractionation of the serum samples using the SP3 protocol followed by LC/MS analysis in data-dependent acquisition mode. An amount of 200 ng was injected on the Evosep LC system equipped with a Pepsep column (15 cm length; ID = 150um) connected to a timsTOF pro mass spectrometer instrument (Bruker Daltonics, Billerica, MA) using the 60 samples per day. The data were searched using MSFragger software v3.1.1 using the following parameters: a maximum of three missed cleavages, oxidation of methionine residues set as variable modification and carbamidomethylation of cysteine residues set as fixed modification (other parameters were set as default).

### Data analysis

The data were then searched to obtain the protein identification and quantification in all samples. RStudio software packages were utilized for all statistical analyses (https://www.rstudio.com). In the current report, our statistical analyses were focused on determining how levels of various proteins were associated with clinical pain severity, ESR, and clinical severity (as determined by cJADAS), and also, whether and how identified pain-associated proteins were integrated with other elements of the JIA (i.e., inflammation, clinical severity and CNS properties) Spearman correlation coefficients were calculated to determine correlations between protein density and clinical pain severity, while Pearson’s correlation coefficients were calculated to determine significant associations among continuous variables (e.g., protein levels, ESR values, and CNS morphological properties). Furthermore, as clinical pain severity is a subjective measure and clear cut off points are ill-defined, we aimed to investigate the serum proteome changes related to self-reports of pain using a more unbiased statistical approach by taking the extremes (i.e., the samples with the lowest and highest scores were sorted into two main groups generating “Low” and “High” pain JIA cohorts). Subsequently, a statistical comparison was performed using a student t-test comparing the mean difference between the High and Low pain cohorts. The results were shown by a Volcano plot with the log2 mean difference on the x-axis and the -log10 *p*-value on the y-axis.

## Results

### Patient overview

Clinical pain intensity ratings of the enrolled JIA patients ranged from 0 to 8 (mean pain intensity rating: 3.00 ± 2.56). Furthermore, ESR values ranged from 1 to 26 (mean ESR: 10.92 ± 8.54) and cJADAS scores were between 0 and 16 (mean score: 5.17 ± 5.78).

### Identification of pain-associated proteins using mass spectrometry analysis

**Fig. 1:**
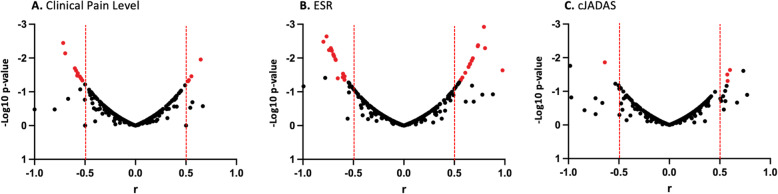
Correlation Between Protein Expression and Clinical Benchmarks. Volcano plots of protein intensity correlation to **(A)** pain severity, **(B)** erythrocyte sedimentation rate (ESR) values and (C) clinical juvenile disease activity score (cJADAS). Significantly (*p* < 0.05) correlated proteins are plotted in red. Proteins passing an r value of 0.5, but not significant due to the small number of data points are shown in black. Further details on proteins significantly correlating with clinical pain levels are provided in Table 1

**Fig. 2:**
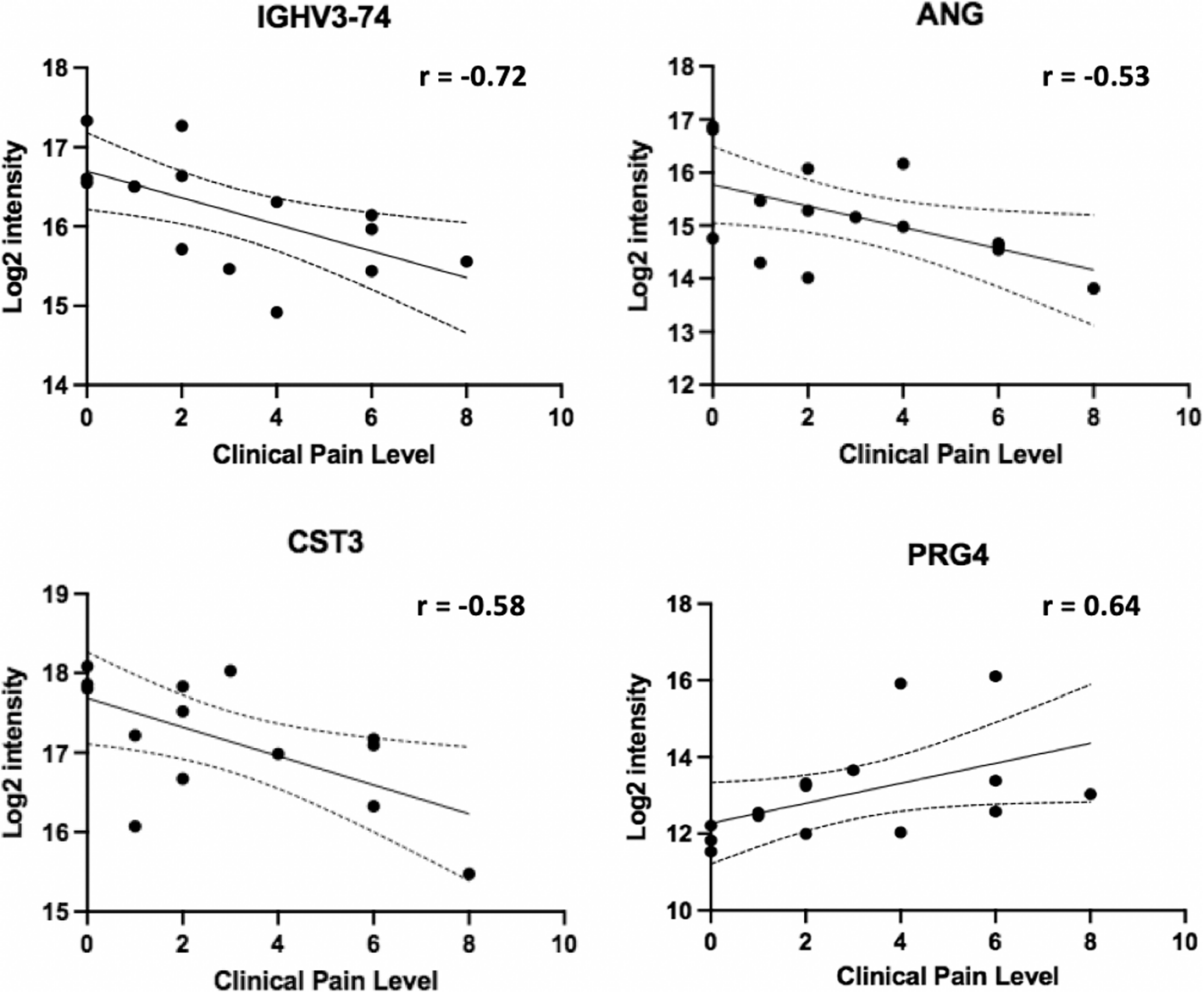
Association Between Protein Expression and Clinical Pain Severity. Spearman correlation analyses were performed to determine the relationship between protein intensities and self-reported clinical pain severity. 95% confidence interval and Spearman correlation value (r) are depicted in each correlation plot. Proteins spanning distinct functional roles are depicted (i.e., IGHV3.74 (humoral immunity); ANG (angiogenesis); CST3 (inflammatory regulation); and PRG4 (articular joint, boundary lubrication)

**Fig. 3:**
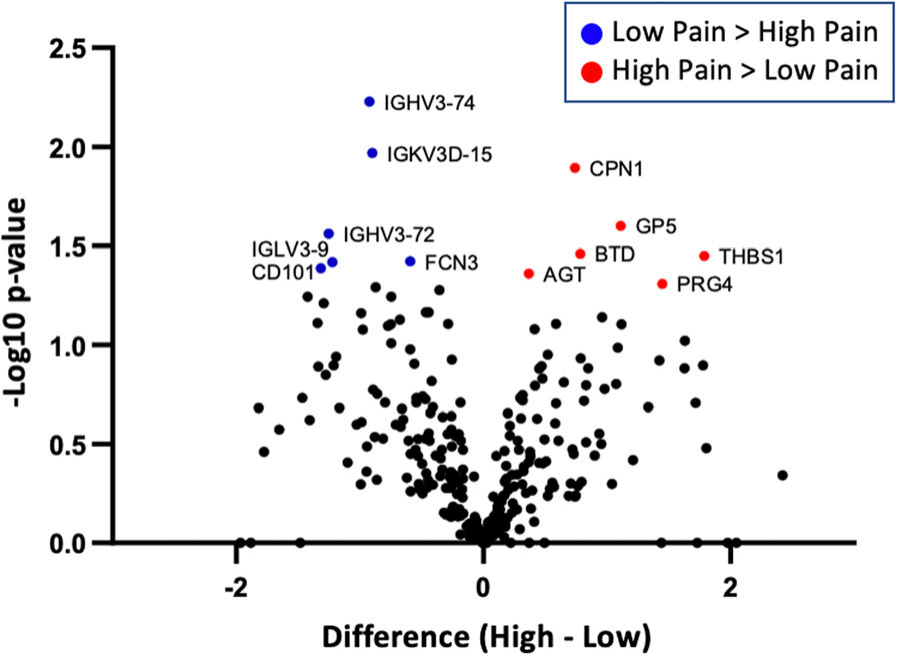
Serum proteomics comparison between low and high pain JIA patients. Sample grouping using scores of 0, 1, and 2 for the low pain cohort, and scores of 4, 6, and 8 for the high pain cohort was used. A comparison of protein expression was performed between low and high pain extremes or sub-cohorts of JIA patients. Blue data points represent proteins significantly (*p* < 0.05) greater for the low pain group, while red data points represent proteins significantly greater for the high pain group

High throughput serum proteomics of the samples collected from 15 JIA patients identified 306 proteins. Only proteins detected in 9 or more patients were used for further analyses. Spearman’s correlation analyses between protein expression and clinical pain severity scores resulted in 14 protein that showed positive (r > 0.5) or negative (r < − 0.5) correlations with pain levels (Table 1, Fig. 1**A)**. Functional roles of the 14 proteins ranged from humoral immunity, angiogenesis, autoimmunity and enzymatic reactions (Table 1, Fig. 2). Additionally, protein expression was evaluated using a grouping method based on the pain scale 0, 1, and 2 to define a ‘Low’ pain group and 4, 6, and 8 to define ‘High’ pain group (Fig. 3). This grouping for ‘Low’ and ‘High’ demonstrated a number of 12 differentially expressed proteins (DEPs; *p* < 0.05).

**Table 1:** Proteins vs. Clinical Pain Level. Proteins identified in serum of JIA patients demonstrating significant (*p* < 0.05) association to clinical pain levels with corresponding function. Samples sizes of *N* = 14* or *N* = 15** were present for all proteins

Proteins Related to Pain	Function	Spearman Correlation Coefficient (r)	P-value (two-tailed)	95% confidence interval
IGLV3.9*	Immunoglobulins: Humoral immunity	−0.70	0.007	−0.90 to − 0.25
IGHG4**	Immunoglobulins: Humoral immunity	−0.60	0.020	−0.86 to − 0.11
IGKV1.5**	Immunoglobulins: Humoral immunity	0.54	0.040	−0.83 to − 0.023
IGKV3D.15**	Immunoglobulins: Humoral immunity	0.59	0.024	−0.85 to − 0.091
IGHV3.72**	Immunoglobulins: Humoral immunity	−0.54	0.041	−0.83 to − 0.018
IGHV3.74	Immunoglobulins: Humoral immunity	−0.72	0.004	−0.902 to − 0.31
LGALS3BP**	Promotes integrin-mediated cell adhesion: Immune response	0.52	0.049	−0.005 to 0.82
ANG**	Role in angiogenesis and autoimmune response, suggested to play a role in immune-mediated inflammatory response	−0.53	0.046	−0.82 to − 0.002
PRG4**	Role in boundary lubrication within articulating joints, involved in vesicle-mediated transport in immune responses	0.65	0.011	0.183 to 0.874
CST3*	Cystatin C: Inhibition of cysteine proteases, involved in inflammation and immune regulation, vascular remodeling and cell migration	−0.58	0.033	−0.85 to − 0.050
VWF**	Hemostasis, thrombosis and vascular inflammation	−0.55	0.035	−0.84 to − 0.038
ALB**	Regulate osmotic pressure	0.57	0.028	−0.84 to − 0.067
AGT**	Angiotensinogen: Blood pressure and fluid and salt regulation	0.55	0.035	0.041 to 0.84
BTD**	Enzyme biotinidase: Biotin removal from food	0.53	0.045	0.005 to 0.824

### Relationship between pain-associated proteins and ESR values and cJADAS scores

Amongst all 306 proteins identified in the serum of JIA patients, various proteins were associated with ESR (Fig. 1**B,** see also **Supplemental Fig.** **1****)**. Table 2 denotes the level of association among Pain-associated proteins and ESR levels. IGLV3.9, PRG4, CST3, VWF and ALB in particular showed significant correlation with ESR with most proteins having roles in immune-mediated responses in addition to other cellular functions (e.g., vascular remodeling and cell migration (CST3) or boundary lubrication within articulating joints (PRG4)). Significant correlation between cJADAS scores and protein levels were found for IGHV6–1 (r = − 0.64, *P* = 0.014), IGHV2–70 (r = 0.58, *P* = 0.05), IGHD (r = 0.60, *P* = 0.023), and IGLC3 (r = 0.57, *P* = 0.03) **(**Fig. 1**C)**, which were primarily related to humoral immunity [29]. These correlation values listed above were derived based on 14 data points. A significant association between Pain-associated proteins and cJADAS scores were absent throughout all Pain-associated proteins (Table 2).

**Table 2:** Pain-associated Proteins vs. ESR and cJADAS. Pain protein (see Table 1) correlation with erythrocyte sedimentation rate (ESR) and clinical juvenile disease activity score (cJADAS) in JIA patients. Correlation (r) and P-values (two-tailed) are displayed. Sample sizes (N) are denoted for each correlation analyses

Pain Proteins	Correlation with ESR (*N* = 13)	Correlation with cJADAS (*N* = 14)
IGLV3.9	**r = − 0.59,** ***P*** **= 0.04**	r = − 0.34, *P* = 0.26 (*N* = 13)
IGHG4	r = − 0.10, *P* = 0.76	r = − 0.49, *P* = 0.079
IGKV1.5	r = − 0.27, *P* = 0.37	r = − 0.16, *P* = 0.60
IGKV3D.15	r = 0.001, *P* = 0.99	r = 0.011, *P* = 0.97
IGHV3.72	r = − 0.49, *P* = 0.09	r = 0.12, *P* = 0.685
IGHV3.74	r = − 0.30, *P* = 0.31	r = − 0.02, *P* = 0.95
LGALS3BP	r = 0.20, *P* = 0.51	r = 0.31, *P* = 0.28
ANG	r = 0.02, *P* = 0.95	r = − 0.36, *P* = 0.21
PRG4	**r = 0.65,** ***P*** **= 0.02**	r = − 0.27, *P* = 0.35
CST3	**r = − 0.74,** ***P*** **= 0.01**	r = − 0.18, *P* = 0.56 (*N* = 13)
VWF	**r = − 0.72,** ***P*** **= 0.01**	r = 0.10, *P* = 0.73
ALB	**r = − 0.70,** ***P*** **= 0.01**	r = 0.09, *P* = 0.75
AGT	r = 0.16, *P* = 0.59	r = − 0.02, *P* = 0.95
BTD	r = 0.13, *P* = 0.66	r = 0.05, *P* = 0.86

### Relationship between pain-associated proteins and central morphological properties

Our recent investigation utilizing the same JIA patient sample identified a significant association between caudate volume and clinical pain intensity ratings as well as a significant correlation between cortical thickness of the insula cortex and ESR values [27]. Therefore, the relationship, or lack thereof, between Pain-associated proteins described above and morphological properties of the caudate nucleus and insula were explored.

The left hemisphere caudate nucleus volume was not significantly correlated with any of the Pain-associated proteins (Table 3). The right hemisphere caudate nucleus volume negatively correlated with BTD and AGT, while positively correlated with IGHV3.74. The left insular cortical thickness was not significantly correlated to any of the Pain-associated proteins. However, significant (*P* = 0.05) associations were revealed between right insular thickness values and two of the Pain-associated proteins (BTD and LGALS3BP).

**Table 3:** Pain-associated Proteins vs. Central Morphological Properties. Pain protein (see Table 1) correlation with caudate nucleus volume and insular thickness values in JIA patients. Pearson correlation r values and corresponding *P*-values (two-tailed) are displayed. Sample size is indicated for each correlation pair

Pain Proteins	Left Caudate Nucleus Volume (*N* = 9)	Right Caudate Nucleus Volume (*N* = 9)	Left Insular Thickness (*N* = 9)	Right Insular Thickness (*N* = 9)
IGLV3.9	r = 0.56, *P* = 0.12	r = 0.64, *P* = 0.06	r = 0.11, *P* = 0.77	r = 0.54, *P* = 0.14
IGHG4	r = 0.41, *P* = 0.27	r = 0.30, *P* = 0.43	r = 0.05,*P* = 0.90	r = 0.11, *P* = 0.77
IGKV1.5	r = 0.13, *P* = 0.75	r = 0.27, *P* = 0.49	r = 0.27, *P* = 0.48	r = 0.43, *P* = 0.24
IGKV3D.15	r = 0.58, *P* = 0.10	r = 0.64, *P* = 0.06	r = − 0.25, *P* = 0.51	r = 0.31, *P* = 0.42
IGHV3.72	r = 0.41, *P* = 0.28	r = 0.435, *P* = 0.242	r = 0.15, *P* = 0.70	r = 0.14, *P* = 0.72
IGHV3.74	r = 0.63, *P* = 0.07	**r = 0.70,** ***P*** **= 0.04**	r = − 0.13, *P* = 0.73	r = 0.52, *P* = 0.15
LGALS3BP	r = −0.63, *P* = 0.07	r = − 0.64,*P* = 0.07	r = − 0.61, *P* = 0.09	**r = − 0.75,** ***P*** **= 0.02**
ANG	r = 0.44, *P* = 0.24	r = 0.44, *P* = 0.23	r = 0.47, *P* = 0.19	r = 0.54, *P* = 0.14
PRG4	r = − 0.53, *P* = 0.14	r = − 0.52, *P* = 0.15	r = − 0.25, *P* = 0.51	r = − 0.24, *P* = 0.53
CST3	r = − 0.02, *P* = 0.95	r = 0.03, *P* = 0.95	r = 0.65, *P* = 0.06	r = 0.28, *P* = 0.47
VWF	r = 0.30 *P* = 0.43	r = 0.33, *P* = 0.39	r = 0.36, *P* = 0.34	r = 0.17, *P* = 0.67
ALB	r = 0.57, *P* = 0.11	r = 0.62, *P* = 0.07	r = 0.13, *P* = 0.74	r = 0.26, *P* = 0.50
BTD	r = − 0.67, *P* = 0.051	**r = − 0.78,** ***P*** **= 0.01**	r = − 0.17, *P* = 0.66	**r = − 0.69,** ***P*** **= 0.04**
AGT	r = −0.61, *P* = 0.082	**r = − 0.73,** ***P*** **= 0.03**	r = 0.15, *P* = 0.70	r = − 0.45, *P* = 0.22

## Discussion

There is a clear need for a deeper understanding of the complex mechanisms that underlie pain in JIA and other rheumatic conditions impacting pediatric populations. The current study revealed a number of Pain-associated proteins by characterizing the serum proteome derived from JIA patients. The identified proteins ranged in their biological functions from humoral immunity, angiogenesis, autoimmunity and enzymatic reactions. Furthermore, these preliminary results further suggest the close interactions between immune and nervous systems, and the critical role immune cells and their mediators play in regulating acute and chronic pain states [30].

Many of the identified Pain-associated proteins are involved in immune processes. LGALS3BP is a heavily glycosylated secreted molecule with an immunoinhibitory function [31] often found to be upregulated in cancer, but has also been implicated in various other diseases (e.g., RA) [32]. Furthermore, the subvariants IGLV3.9, IGHG4, IGKV1.5, IGKV3D.15, IGHV3.72 and IGHV3.74 are all members of the immunoglobulin family, binding to particular antigens as part of the immune response, but specific targets of these immunoglobulins are unknown [33]. The identification of immunoglobulins whose abundance levels show positive correlations with pain severity is consistent with current experimental therapies, as clinical trials on binding immunoglobulin protein (BiP), targeting immunoglobulins, have been performed for treatment of RA [34]. Amongst an abundance of processes, CST3 is also considered to be involved with immune responses [35]. ANG, or angiogenin, is most often implicated in tumor-associated angiogenesis, but has been suggested to inhibit inflammatory processes [36, 37] and to mediate local inflammation in arthritis [38]. Moreover, VWF is thought to reflect vascular damage and thrombosis [39]. Lastly, PRG4 has been shown to play a protective factor within articular joints [40], but its role in mediating anti-inflammatory processes has also been confirmed [41]. The association that is found between the density of these predominantly immune related proteins in serum and clinical pain severity in JIA patients, could reflect inflammatory and immune components of pain in JIA. Furthermore, five proteins (IGHV3.9, PRG4, VWF, ALB and CST3) were significantly associated with ESR values, with CST3, a protein active in neurodegenerative (Alzheimer’s Disease) and demyelinating (multiple sclerosis) neurological conditions [42]. ESR values obtained from blood samples reflect overall inflammatory status, and do not capture isolated joint inflammation. Thus, biological samples derived from the synovial compartment of inflamed joints may better identify proteomic markers that are more closely associated with joint inflammation and inflammatory pain.

The current sample consisted of patients who were in remission, reported pain without joint inflammation or reported pain with joint inflammation, where inflammatory status and other joint-related pathology was determined via musculoskeletal MRI. Moreover, our study confirms that pain in JIA is not always commensurate with the amount of inflammation, and also, subsets of proteins were solely associated with inflammation, but not pain. While confirmation in larger studies is necessary, we hypothesize that expression of some proteins is driven by pain and likely associated with peripheral or central sensitization, and other proteins will track more with the inflammatory status of JIA patient or JIA sub-type.

A finer assessment of Pain-associated proteins in JIA is arguably obtained by differentiating patients based on additional diagnostic criteria, which include, JIA subtype (e.g., polyarticular JIA vs. systemic JIA), rheumatoid factor (RF) status, or antinuclear antibody (ANA) status as each can be associated with distinct JIA disease trajectories or severity [43]. A more specific, phenotypic differentiation of JIA patients may elucidate differential proteins expression of proteins in conjunction with distinct sets of Pain-associated proteins. Likewise, comparison of patients based on treatment regimen (e.g., methotrexate vs. methotrexate + TNF-α inhibitor) may also yield unique proteomic signatures. Although this pilot cohort varied in terms of JIA subtype, RF and ANA status, or treatment type the limited sample size did not allow for a robust comparison across these domains. Future work will investigate the contribution of these JIA subtypes and phenotypes in a broader population and importantly include clinical control groups. The incorporation of control cohorts is essential in order to decipher whether proteomic alterations are disease specific for JIA or pain specific.

Previous work aimed to gain more insight into the proteomic underpinnings of pain by performing a meta-analysis of 535 pain related genes in the human cerebrospinal fluid (CSF) [44]. These genes are typically responsible for proteins related to synaptic transmission, inflammatory responses, neuropeptide signaling, and hormonal activity. Furthermore, this study pointed to ten proteins that were potential factors for distinguishing dysfunctional (fibromyalgia) from inflammatory pain (rheumatoid arthritis) disorders. Although proteomic composition in the CSF will differ from blood serum, both tissues can be used to identify Pain-associated proteins. Considering the age of the cohort of JIA patients in this study, retrieving CSF samples was not feasible for ethical reasons. Serum protease network behavior in complex regional pain syndrome (CRPS) has also been recently investigated [45, 46]. Here, relative to controls and other clinical pain populations, CRPS patients showed distinct degradation activity of inflammatory mediators that likely play a role in the development of post-traumatic pain. Konig and colleagues have specifically suggested that degradation of dabsyl-bradykinin is particularly compromised in CRPS, which subsequently drives an inflammatory process.

In prior work, JIA patients were shown to harbor altered CNS properties. Specifically, caudate nucleus volume was negatively correlated to clinical pain intensity and decreased cortical thickness of the insula was not only observed to be lower in JIA patients relative to controls, but also showed a negative correlation with ESR values [27]. Besides a role of the caudate nucleus in the motor system, it has also been proposed to play an important part in modulation of pain experience [47]. The insula, a key hub of the salience networks, plays an essential role in the regulation of emotional aspects of pain [48]. Correlations between both left and right caudate nucleus volume and some of the proteins were observed, and HRG was associated with caudate nucleus volume bilaterally and with right insular thickness. Left insular thickness did not correlate with any of the identified pain related protein densities, whilst right insular thickness was associated with three of the proteins, BTD, LGALS3BP and HRG. The associations of morphological properties and pain related proteins points to an interplay between peripheral mechanisms and the CNS. Pain in JIA is multifaceted and could be underpinned by altered proteomic composition contributing to pain and altered neurobiological properties to the emotional mediation of this pain.

With the novelty of this investigation into proteomic alterations concerning pain in JIA, limitations of this exploratory study are noted. Mainly, the small sample size was a limiting factor in this investigation yet provides a basis for extending this work into a larger patient sample. Also, all patients were on active treatment during study evaluation, and treatment plans varied from single immune-modulatory agents (i.e., methotrexate or adalimumab) to combination therapies (i.e., methotrexate and adalimumab). Moreover, some patients also noted intermittent use of non-steroidal anti-inflammatory drugs (e.g., naproxen). The type of therapy could have impacted the serum proteomic composition, particularly in specific immune-related proteins in the collected serum. In order to determine the influence of various therapies on the JIA proteome, future studies should characterize protein expression before and after treatment or before and after the onset of a new therapeutic regimen. Characterization of the proteome at multiple points post-treatment induction may also provide new biological insights towards treatment response vs. non-response.

## Conclusions

In summary, LC/MS-based serum proteomic analysis identified 14 Pain-associated proteins in the serum of JIA patients. A number of the identified proteins had functional roles in immune or inflammatory processes. This investigation provides novel insights into deviations in the proteome in relation to clinical pain in JIA patients, and could contribute to the ongoing search for prognostic markers and treatment targets for JIA pain.

## Supplementary Information

**Additional file 1.**

## Data Availability

Data are available from the corresponding authors upon reasonable request and with permission of all sites contributing data.

## Abbreviations

AI: Anterior insula
ANA: antinuclear antibodies
BCH: Boston Children’s Hospital
BiP: binding immunoglobulin protein
cJADAS: Clinical Juvenile Disease Activity Score
CNS: Central nervous system
CSF: cerebrospinal fluid
DMARDs: Disease-modifying anti-rheumatic drugs
ESR: Erythrocyte sedimentation rate
fMRI: Functional magnetic resonance imaging
IL: interleukin
JIA: Juvenile idiopathic arthritis
LC: liquied chromatography
MRI: magnetic resonance imaging
MS: mass spectrometry
MIF: macrophage inhibitory factor
PROMIS: Patient-Reported Outcomes Measurement Information System
RF: rheumatoid factor
SD: Standard deviation
RA: Rheumatoid arthritis
RF: Rheumatoid factor
TNF-α: Tumor Necrosis Factor-α
