# Supplementary material for: Proteomics based markers of clinical pain severity in juvenile idiopathic arthritis
Source: Pediatr Rheumatol Online J. 2022 Jan 15;20:3. doi: 10.1186/s12969-022-00662-1 (PMC8761318; doi:10.1186/s12969-022-00662-1)
Supplement: Supplementary file 1 — Additional file 1. [file 12969_2022_662_MOESM1_ESM.docx]

**Supplemental Document**

**Proteomics Based Markers of Clinical Pain Severity in Juvenile Idiopathic Arthritis**

*Hanne Van Der Heijden^1,2,3#^, Benoit Fatou^4#^, Diana Sibai^1^*, *Kacie Hoyt^5^, Maria Taylor^5^, Kin Cheung*^6^*, Jordan Lemme^1^, Mariesa Cay^1^, Benjamin Goodlett^7^, Jeffery Lo^5^, Melissa M. Hazen^5^, Olha Halyabar^5^, Esra Meidan^5^, Rudy Schreiber^2^, Camilo Jaimes^7^, Kirsten Ecklund^7^, Lauren A. Henderson^5^, Margaret H. Chang^5^, Peter A. Nigrovic^5^, Robert P. Sundel^5^, Hanno Steen^4,9,10*^, Jaymin Upadhyay^1,11*^*

^1^ Department of Anesthesiology, Critical Care and Pain Medicine, Boston Children’s Hospital, Harvard Medical School, Boston, MA USA

^2^ Faculty of Psychology and Neuroscience, Section Neuropsychology & Psychopharmacology

Maastricht University, Maastricht, The Netherlands.

^3^ Faculty of Science, Biomedical Sciences Neurobiology, University of Amsterdam, Amsterdam, The Netherlands

^4^ Department of Pathology, Boston Children's Hospital, Harvard Medical School, Boston, MA.

*^5^* Division of Immunology, Boston Children's Hospital, Harvard Medical School, Boston, MA, USA

^6^ BioSAS Consulting, Inc. Wellesley MA USA.

^7^ Division of Genetics and Genomics, Boston Children’s Hospital, Harvard Medical School, Boston, MA USA

^8^ Department of Radiology, Boston Children’s Hospital, Harvard Medical School, Boston, MA USA

^9^ Neurobiology Program, Boston Children’s Hospital, Boston, MA USA

^10^ Precision Vaccines Program, Boston Children’s Hospital, Boston, MA USA

^11^ Department of Psychiatry, McLean Hospital, Harvard Medical School, Belmont, MA USA

**Supplemental Table 1: Patient characteristics**

| **Patient** | **Age**  **(y)** | **Gender** | **JIA**  **Subtype** | **Medication**  **Usage** |  | **Pain Level**  **(Patient Reported)** | **ESR, mm/hr** |  | **cJADAS** | |  |
| --- | --- | --- | --- | --- | --- | --- | --- | --- | --- | --- | --- |
| 1 | 10 | M | oJIA, ANA- | Methotrexate |  | 6 | 3 |  | | 12 | |
| 2 | 13 | M | JPsA, ANA- | Methotrexate, Secukinumab |  | 1 | 17 |  | | 2 | |
| 3 | 9 | F | pJIA, ANA+ | Methotrexate, Adalimumab |  | 0 | --- |  | | 5 | |
| 4 | 15 | F | pJIA, ANA+,  RF- | Methotrexate, Infliximab |  | 2 | 6 |  | | 16 | |
| 5 | 16 | F | pJIA, RF+ | Adalimumab |  | 8 | 20 |  | | 12 | |
| 6 | 12 | M | pJIA, RF+ | Methotrexate, Adalimumab |  | 6 | 23 |  | | 1 | |
| 7 | 16 | F | pJIA, RF-, ANA+ | Methotrexate, Infliximab |  | 0 | 8 |  | | 1 | |
| 8 | 8 | M | pJIA, RF- | Methotrexate, Adalimumab |  | 4 | 14 |  | | 0 | |
| 9 | 13 |  | pJIA, RF- | Methotrexate, Adalimumab |  | 2 | 2 |  | | --- | |
| 10 | 12 | F | pJIA, ANA+, RF- | Leflunomide |  | 3 | 2 |  | | 1 | |
| 11 | 9 | F | oJIA, ANA- | Methotrexate, Adalimumab |  | 1 | 7 |  | | 0 | |
| 12 | 16 | F | pJIA, ANA+, RF- | Adalimumab |  | 6 | 13 |  | | 10 | |
| 13 | 10 | F | pJIA, RF+ | Methotrexate, Adalimumab |  | 2 | --- |  | | 4 | |
| 14 | 13 | F | pJIA, RF- | Methotrexate |  | 0 | 1 |  | | 0 | |
| 15 | 15 | F | pJIA, RF-ANA+ | Methotrexate |  | 4 | 26 |  | | 0 | |

**ESR**: erythrocyte sedimentation rate

**cJADAS:** clinical Juvenile Disease Activity Score:

**JIA:** juvenile idiopathic arthritis

**oJIA:** oligoarticular juvenile idiopthic arthritis

**JPsA:** juvenile psoriatic arthritis

**pJIA:** polyarticular JIA

**ANA:** antinuclear antibody

**psJIA:** psoriatic

**RF:** rheumatoid factor

**
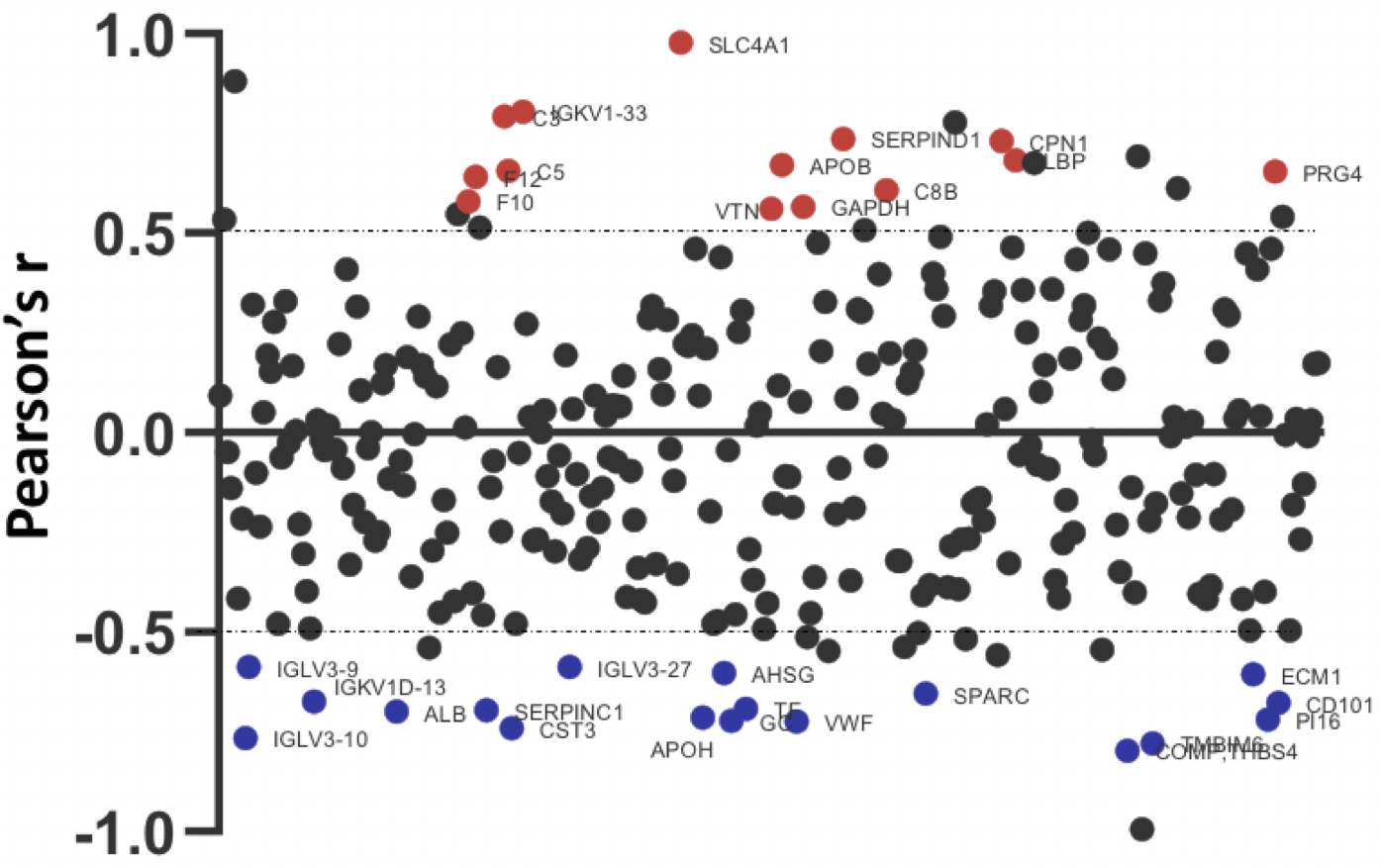
**

**Supplemental Figure 1.** **Association Between** **Protein Expression and Erythrocyte Sedimentation Rate (ESR).** Pearson correlation analysis between the individual protein intensities and ESR values. The proteins highlighted in red and blue correspond to the significant (p< 0.05) positively and negatively correlated proteins, respectively. Proteins passing an r value of 0.5, but not significant due to the small number of data points are shown in black.
